# Better Brain and Cognition Prior to Surgery Is Associated With Elevated Postoperative Brain Extracellular Free-Water in Older Adults

**DOI:** 10.3389/fnagi.2019.00117

**Published:** 2019-05-16

**Authors:** Jared J. Tanner, Manish Amin, Cheshire Hardcastle, Hari Parvataneni, David E. Vaillancourt, Thomas H. Mareci, Catherine C. Price

**Affiliations:** ^1^Department of Clinical and Health Psychology, University of Florida, Gainesville, FL, United States; ^2^Department of Physics, University of Florida, Gainesville, FL, United States; ^3^Department of Orthopedic Surgery, University of Florida, Gainesville, FL, United States; ^4^Department of Applied Physiology and Kinesiology, Biomedical Engineering, and Neurology, University of Florida, Gainesville, FL, United States; ^5^Department of Biochemistry and Molecular Biology, University of Florida, Gainesville, FL, United States; ^6^Department of Anesthesiology, University of Florida, Gainesville, FL, United States

**Keywords:** diffusion magnetic resonance imaging, cognition, brain, neuroinflammation, total knee arthroplasty, anesthesia

## Abstract

For adults age 65 and older, the brain shows acute functional connectivity decreases after total knee arthroplasty with the severity of change predicted by preoperative cognitive function and brain disease burden. The extent of acute structural microstructural brain changes acutely after surgery remains unknown within the literature. For the current study, we report on the severity of acute post-surgery microstructural brain changes as measured by diffusion imaging and free-water analysis. Participants who underwent total knee arthroplasty under general anesthesia and non-surgery peers were part of a federally funded prospective cohort investigation involving participants. Recruitment occurred between 2013 and 2017. Data were collected in outpatient and inpatient settings within a university-affiliated medical center. A total of 232 TKA patients were referred by the study surgeon and contacted for study inclusion. Of these, 78 met inclusion and exclusion criteria and completed assessment. Five participants were excluded due to anesthetic protocol changes (spinal instead of general) with an additional 12 excluded for imaging-related complications. The total included sample size was 61. A total of 127 non-surgery participants were screened with 66 enrolled. One non-surgery participant was excluded for an imaging-related complication. Total knee arthroplasty and general anesthetic protocols were standardized. Participants received preoperative neurocognitive assessment and brain magnetic resonance imaging, with repeat imaging 48 h after surgery or pseudo surgery. Free-water analyses were performed using diffusion weighted images and tract-based spatial statistics with baseline cognitive data used to predict free-water changes. Surgery participants had widespread increases in white matter free-water. Surgery participants with higher cognitive functions as measured by immediate memory and less evidence of brain atrophy and disease (i.e., brain integrity) had greater free-water increase. Non-surgery peers had no free-water change. We interpret the surgery group’s free-water change as indicating widespread brain white matter glial response, with greater change indicative of better brain response to the acute surgery/anesthesia experience.

## Introduction

Coupled with the exponential growth of older adults is an increased need or desire for operative interventions that include anesthesia. This is concerning for two reasons: (1) Older age is a risk factor for negative postoperative outcomes (Moller et al., 1998; Monk et al., 2008) and (2) mechanisms for negative outcomes remain poorly understood. Based on integrated theories of brain reserve and diathesis stress models (Belsky and Pluess, 2009), some researchers postulate that patients’ preoperative cognitive and brain integrity are important considerations to understanding mechanisms of vulnerability (Goto et al., 2001; Price et al., 2014, 2017).

While functional MRI network connectivity decline is reported by two separate research teams for cardiac surgery (Browndyke et al., 2017) and orthopedic surgery (Huang et al., 2018), much less information is known about acute microstructural changes in older adult brains after surgery. Diffusion-weighted imaging (DWI) can provide information regarding microstructure. When using the analysis method of free-water imaging, which separates diffusion signals into tissue and extracellular components, it is thought that extracellular free-water volume increases can indicate glial change, including neuroinflammation (Pasternak et al., 2009, 2012, 2015).

The purpose of the present investigation was to examine if free-water brain white matter microstructural changes in a study group occur after the same major surgery with the same anesthesia protocol and if observed changes could be predicted by preoperative brain and cognitive integrity. Using a multimodal, longitudinal imaging protocol with both surgical and control groups, we examined metrics of brain disease burden on free-water change. We hypothesized brain disease burden would explain a portion of free-water change. Disease burden was measured using the severity of leukoaraiosis (LA) as an imaging marker of white matter cerebrovascular disease (Hachinski et al., 1987). Leukoaraiosis is a marker of white matter changes with higher levels associated with reduced executive functions within aging and dementia populations (Price et al., 2012; Gold et al., 2017). Ventricular volume was used as a marker of global white and gray matter atrophy (Apostolova et al., 2013) and entorhinal thickness as a sensitive marker of neurodegenerative processes (Devanand et al., 2007). Neurodegeneration, such as in Alzheimer’s disease, is associated with chronic neuroinflammatory processes and glial activation (for a review see Griffin et al., 1998), which in turn might explain increases seen in brain white matter in older adults with amnestic mild cognitive impairment and Alzheimer’s disease (Ji et al., 2019). Given the dominance of executive/frontal-subcortical functions and memory declines after non-cardiac surgery (Price et al., 2008), aspects of behavioral frontal-subcortical system functions and memory were also examined as potential predictors of microstructural changes.

## Materials and Methods

The University of Florida Institutional Review Board in Gainesville, Florida approved this study. All participants were appropriately informed and signed consents. The study was conducted in accordance to principles of the Declaration of Helsinki.

### Participants

A total of 232 surgery patients were referred by the study surgeon (HP) and contacted for study inclusion. Of these, 92 agreed to consider the study with 78 meeting inclusion and exclusion criteria and completing baseline neuropsychological assessment and MRI. Data from five of the participants were excluded due to anesthetic protocol (spinal instead of general). Data from 12 surgery participants were excluded due to presence of pre-existing silent strokes (1 participant), MRI post-surgery scanner unavailability (2 participants), withdrawal before surgery (1 participant), or incomplete postoperative diffusion data due to pain (8 participants). The total included sample size was 61.

For non-surgery orthopedic peers, a total of 127 participants with self-reported orthopedic pain were recruited from a local orthopedic clinic, community fliers, and targeted mailings. They were screened with 66 enrolled and completed baseline MRI. Non-surgery participants were selected through a yoked review process to match individual surgery participants on age, education, sex, and ethnicity/race. Non-surgery participants had to abstain from surgery for at least 1 year. Both groups were recruited over the same time frame and were tested and scanned at the same time intervals. One participant had an incomplete diffusion sequence, resulting in a total of 65 who completed the baseline assessment and pre-post *pseudo surgery* imaging sessions.

The final participant sample consisted of non-demented individuals over the age of 60 who elected unilateral total knee arthroplasty surgery (TKA; *n* = 61) and non-surgery peers (*n* = 65). Groups matched on age, sex, education years, and race. All participants met the following inclusion/exclusion criteria: (1) aged 60 or older, (2) English as primary language, (3) have osteoarthritis or comparable joint pain, (4) have intact activities of daily living, and (5) have baseline neuropsychological testing unsupportive for dementia criteria per Diagnostic and Statistical Manual of Mental Disorders – Fifth Edition (American Psychiatric Association [APS], 2013). Additional exclusion criteria included: any other major surgery within the study timeline, history of head trauma/neurodegenerative illness, documented learning or seizure disorder, less than a sixth-grade education, substance abuse in the last year, major cardiac disease, chronic medical illness known to induce encephalopathy, implantable device precluding an MRI, and an unwillingness to complete the MRI.

Participants completed a phone cognitive screening (Welsh et al., 1993; Cook et al., 2009) and a comprehensive history/systems interview to confirm inclusion/exclusion criteria, followed by an in-person comorbidity rating (Charlson et al., 1987), activities of daily living (Lawton and Brody, 1969), cognitive testing, and brain MRI. The same examiner completed testing for all participants. Trained raters blind to group condition scored all behavioral data. Two neuropsychologists reviewed the baseline data to confirm test scores met the expected ranges for non-demented individuals.

### Anesthesia and Surgery Protocol

Protocols were standardized, with surgery participants receiving intravenous midazolam (1–4 mg) followed by continuous femoral nerve block (CFNB) and single-injection subgluteal sciatic with 20 mL and 30 mL, respectively, of 0.5% ropivacaine as a bolus injection. The CFNB was continued with ropivacaine 0.2% at an infusion rate of 10 mL per hour. No opioids were added. Propofol, fentanyl, and rocuronium were used for anesthesia induction and intubation. Patients were ventilated with an air/oxygen mixture to maintain an end-tidal carbon dioxide at 35 ± 5 mm; anesthesia was maintained with inhaled isoflurane and intravenous fentanyl and rocuronium.

Total knee replacement surgery was done in a standard manner for all patients by the same surgeon. A tourniquet was used for all cases set to 250 mm Hg and elevated prior to incision and deflated just prior to closure. Bony preparation was done by intramedullary instrumentation for the femoral side and extramedullary for the tibial side. The anterior and posterior cruciate ligaments were sacrificed for all patients and implants were fixed to the bone using bone cement.

Using a published conversion algorithm, morphine equivalent dosages (MED) were calculated for each patient relative to the post-surgery scan (Dowell et al., 2016). The MED was considered potentially active if the most recent dose was within 6 h prior to the post-surgery MRI. Delirium was assessed 24 h postoperatively with the Confusion Assessment Method (Inouye et al., 1990). Pain assessment ratings (0–100; 100 = worst) were acquired during the MRI scan.

### MRI Acquisition

Structural MRI was conducted within 1 week on average before surgery and postoperatively within 48 h after surgery or a pseudo surgery (for non-surgery peers) date. Participants were scanned at two different time points, one corresponding to before surgery and one corresponding to after surgery using a 3.0 T Siemens Verio system with an 8-channel head coil. T1-weighted and diffusion weighted MRI were acquired at both timepoints and T2 FLAIR acquired only at baseline.

T1-weighted images were acquired with the following parameters: TR: 2500 ms; TE: 3.77 ms; 176 sagittal 1 mm^3^ slices, 1 mm isotropic resolution; 256 × 256 × 176 matrix, 7/8 phase partial Fourier. Fluid attenuated inversion recovery (FLAIR) images were acquired with the following parameters: 176 contiguous slices, 1 mm^3^ voxels, TR/TE = 6000/395 ms. Diffusion weighted images were acquired with the following parameters: TR/TE = 17300/81 ms, two scans without diffusion weighting, six diffusion gradient directions with *b*-value of 100 s/mm^2^ and 64 diffusion gradient directions with *b*-value of 1000 s/mm^2^. The diffusion gradients were distributed following a scheme of electrostatic repulsion (Jones et al., 1999). The diffusion-weighted images covered the entire brain with an isotropic resolution of 2.0 mm, field of view (FOV) of 256 mm × 256 mm, and 73 slices.

### MRI Data Processing

Using T1-weighted images, cortical reconstruction and volumetric segmentation was performed with the FreeSurfer image analysis suite (Version 6.0^1^) (Fischl et al., 2002, 2004; Segonne et al., 2004; Fischl, 2012). T1 images were first processed using the cross sectional pipeline and then using the longitudinal FreeSurfer pipeline (Reuter et al., 2012) to calculate ventricle volume, and entorhinal cortex thickness.

#### Diffusion-Weighted Imaging

Translational diffusion structural sequences allow for not only anatomical comparisons, but also measurement of free-water, which separates diffusion signals into tissue and extracellular components (Pasternak et al., 2009, 2012, 2015). The diffusion weighted scans were corrected for eddy current distortion using the FMRIB Software Library (FSL) *eddy_correct* algorithm (Smith et al., 2004; Jenkinson et al., 2012) and gradient vectors rotated after correction. To analyze the changes in water diffusion, a free-water elimination model was used, utilizing an isotropic component for the free water compartment and a single tensor for the more restrictive tissue compartment (Pasternak et al., 2009). The isotropic diffusion was modeled using a free-water diffusivity of 3 × 10^–3^ s/mm^2^, which correlates with CSF diffusion in the brain at body temperature. The anisotropic component was analyzed using FSL’s *dtifit*. Final metrics of interest were: Free-water (FW) and tissue-compartment DTI metrics corrected for free-water: fractional anisotropy (FA), mean diffusivity (MD), radial diffusivity (RD; calculated as the mean of eigenvalues, λ_2_ and λ_3_), axial diffusivity (AD; calculated as eigenvalue λ_1_), λ_2_, and λ_3_.

For both the TKA and non-TKA groups, voxelwise statistical analysis of the FA data was carried out using TBSS (Tract-Based Spatial Statistics; Smith et al., 2006), which is part of FSL. All subjects’ FA data were then aligned into a common space using the non-linear registration tool FNIRT (Andersson et al., 2007), which uses a b-spline representation of the registration warp field (Rueckert et al., 1999). Next, the mean FA image was calculated and then thinned to create a mean FA skeleton, which represents the centers of all tracts common to the group. Each participant’s aligned FA data was then projected onto this skeleton and the resulting data fed into voxelwise cross-subject statistics using *randomise* with 5,000 permutations of the data; a within-group, two-sample paired two-tailed *t*-test was performed, correcting for family-wise errors and applying a threshold-free cluster enhancement. All analyses used scan time of day (Trefler et al., 2016), pain during the MRI, and MED score as covariates. The analyses were then repeated for non-FA images using the script *tbss_non_FA*. We also performed a *post hoc* between group (surgery vs. control) TBSS analysis to assess FW differences at pre-surgery and post-surgery.

In order to assess individual-level predictors of change in diffusion metrics showing significant pre-post changes, group-level *t*-test statistical maps were thresholded at *p* < 0.05 (family-wise error corrected) and converted to binary masks. Binary masks from metrics with widespread significant change were then transformed into individual native diffusion space and mean metric values within the masks were exported for predictive analysis in SPSS (IBM, Version 24).

### Cognitive and Brain Predictors of Interest

#### Current Brain Integrity (BI)

Neuroanatomical brain integrity (BI), prior to the surgery, was evaluated using a composite of (1) total ventricular volume, (2) mean left and right entorhinal cortex thickness, and (3) leukoaraiosis (LA) volume. Larger lateral ventricular volume is a risk factor for dementia and has been used in calculations of brain reserve (Carmichael et al., 2007). Left and right volumes (including temporal horns) were summed to create total ventricular volume. For each participant, total ventricle volume was divided by total intracranial volume (FreeSurfer *maskvol*). The entorhinal cortex is sensitive to neurodegenerative pathology and subsequent cognitive decline (Velayudhan et al., 2013). LA is a term for hyperintense or bright regions within the white matter as seen on T2 magnetic resonance (MR) imaging or hypodense regions on computerized tomography (CT) scans (Hachinski et al., 1987; Black et al., 2009). LA associates with white matter pallor on staining, hyalinosis of the vessels, and atherosclerosis (Jellinger, 2013) and broadly is considered a marker of cerebrovascular disease (Black et al., 2009). Increasing amounts of LA are associated with cognitive declines in healthy non-demented older adults and individuals with dementia (Brickman et al., 2010, 2012; Price et al., 2012).

#### LA Using T2 FLAIR

A reliable rater measured all scans for LA using FLAIR sequences with in-house macros for ImageJ^2^. These semi-automated measurements included periventricular caps and rims and showed criterion validity relative to the Junque LA Scale (Price et al., 2012). LA voxels for each brain slice were thresholded and saved as 2D LA binary masks that were then concatenated into individual 3D binary masks (dice similarity coefficient; DSC = 0.84–0.93; Inter-rater range = 0.80–0.83; DSC mean ± s.d. = 0.84 ± 0.12). Outcome variable = Estimated total brain LA volume in mm^3^.

To create the current BI composite, we calculated *Z*-scores based on all surgery and non-surgery participants. The ventricle and LA *Z*-scores were then multiplied by −1, resulting in higher *Z*-scores implying a more intact brain. The three *Z*-scores were averaged creating the BI composite variable.

#### Premorbid Cognitive Reserve

All baseline measures were acquired within 1 week prior to the surgery/pseudo surgery. Cognitive reserve (CR) is a concept developed to partially explain why there is not a direct relationship between clinical symptoms (e.g., memory) and some factor (e.g., neurodegenerative pathology) that should affect function; higher cognitive reserve is related to psychosocial and experiential factors (e.g., greater educational attainment) and genetic factors (e.g., childhood intelligence) (Stern, 2009). Premorbid cognitive reserve was evaluated as a global composite of three metrics shown to be resistant to neurodegenerative disease processes and are considered estimates of premorbid intelligence (Lezak, 2012): word reading ability [Wide Range Achievement Test (Wilkinson and Robertson, 2006); total words correctly read], vocabulary knowledge [Wechsler Abbreviated Scale of Intelligence; Holdnack (1999); WASI; Vocabulary Subtest], and years of education. We hypothesized CR would explain a portion of free-water changes.

#### Preoperative Cognitive Domains of Interest

We completed a comprehensive cognitive assessment to address the *a priori* hypothesis that preoperative frontal-subcortical functions (e.g., working memory and immediate recall) would explain a portion of pre- to post-surgery structural changes. We tested hypotheses regarding frontal lobe mediated behavior (attention, working memory, and immediate recall) versus temporal lobe mediated behavior (retention and delay recall with relatively weaker working memory and attention components). However, both composites involve measures assessing wide brain networks of bilateral white and gray matter (Jeneson and Squire, 2012) so both composite scores might relate to any white matter changes. The composites are described below.

##### Frontal-subcortical functions

Hopkins verbal learning test – revised (HVLT) (Brandt and Benedict, 2001) trial 1 total words recalled, Rey–Osterrieth complex figure (ROCF) (Osterrieth, 1944) immediate recall total score, and digit span backward (Wechsler adult intelligence scale – III) (Holdnack, 1997a), longest backward span. The ROCF immediate recall was performed without a delay after the copy condition.

##### Delay memory (DM)

Logical Memory II (delay recall, retention, and recognition scores) from the Wechsler memory scale, 3rd edition (WMS-III) (Holdnack, 1997b), Hopkins verbal learning test – revised (HVLT) delay recall, retention, and recognition (Benedict et al., 1998), and ROCF delay recall total score (Osterrieth, 1944).

### Statistical Analyses

We assessed between-group differences in age, education, premorbid estimated intelligence, days between MRI scans, general cognition (MoCA), and pain during the MRI using *t*-tests. To assess microstructural white matter changes, we used Pearson product moment correlations to examine associations between cognitive and brain free-water and DTI independent variables of interest. We then used a hierarchical linear regression analysis to examine the combined and proportional variance in free-water change explained by brain integrity, cognitive reserve, frontal-subcortical functions, and declarative memory. Age and race are both associated with systemic inflammation (Lynch, 2010; Stepanikova et al., 2017) so their effects were controlled for in the regression models to account for possible interactions with free-water change. Age and race were entered as Model 1, cognitive reserve was added for Model 2, brain integrity was added for Model 3, frontal-subcortical functions was added for Model 4, and delay memory was added for Model 5. This approach was repeated for MD, AD, and RD dependent variables.

## Results

### Participant Characteristics

The final groups of participants (surgery *n* = 61; non-surgery *n* = 65) were not statistically different in age or years of education (Table 1). There was no statistical group difference in baseline pre-surgery pain level while in the MRI scanner; however, post-surgery comparisons showed the surgery group had significantly higher pain than non-surgery peers (*p* < 0.05). Days between baseline/pre-surgery MRI and post-surgery MRI were not statistically different by group (*p* = 0.18). Although four surgery participants were identified with delirium lasting less than one day no participants had evidence of delirium at the time of the post-surgery MRI; all participants were included in analyses. Surgery participants had lower cognitive reserve, frontal-subcortical functions, and delay memory than control participants (*p*-values < 0.05) but did not differ in brain integrity (Table 1). One surgery participant had an acute, focal right inferior parietal white matter stroke that appeared on the post-surgery MRI. There were no reported symptoms with the stroke.

**TABLE 1:** Demographic, brain, and cognitive scores by group.

	TKA	Non-TKA
	(*n* = 61)	(*n* = 65)
Age	69.13±7.02,60-85	68.63±5.59,60-83
Education	15.36±2.85,10-23	16.05±2.65,9-24
Race (Black:White)	8:53	4:61
PreMRI pain	10.78±18.14,0-75	8.12±15.47,0-70
PostMRI pain**	38.67±23.53,0-100	7.60±10.27,0-40
MED	11.97±10.99,0-37.50	–
Brain integrity	-0.05±0.74,-2.05-1.07	0.04±0.62,-1.79-1.14
Cognitive reserve**	0.34±0.63,-0.84-1.84	0.69±0.56,-1.20-1.88
Processing speed**	-0.04±0.54,-1.27-1.29	0.26±0.68,-1.74-1.84
Immediate memory**	-0.36±0.74,-1.90-1.20	0.10±0.88,-1.65-2.02
Delay memory**	-0.21±0.79,-2.00-1.06	0.21±0.71,-2.17-1.40

**p < 0.01. PreMRI Pain, participant-reported pain (0–100 scale) during the baseline MRI; PostMRI Pain, participant-reported pain (0–100 scale) during the follow-up MRI; MED, morphine equivalent dose. Brain integrity is a *z*-score composite of ventricle volume, leukoaraiosis volume, and entorhinal cortex thickness. Cognitive reserve is a composite of word reading ability, vocabulary knowledge, and years of education. Processing speed is a composite of stroop word reading total correct in 45 s, digit symbol raw score in 120 s, trail making test, Part A total time in seconds. Immediate memory is a composite of Hopkins verbal learning test – revised (HVLT) trial 1 total words recalled and Rey–Osterrieth complex figure immediate recall. Delay memory is a composite of Hopkins verbal learning test – revised (HVLT) delay recall and retention and Rey–Osterrieth complex figure delay recall.

### Neuroimaging

#### Free-Water and DTI Analyses

**FIGURE 1:**
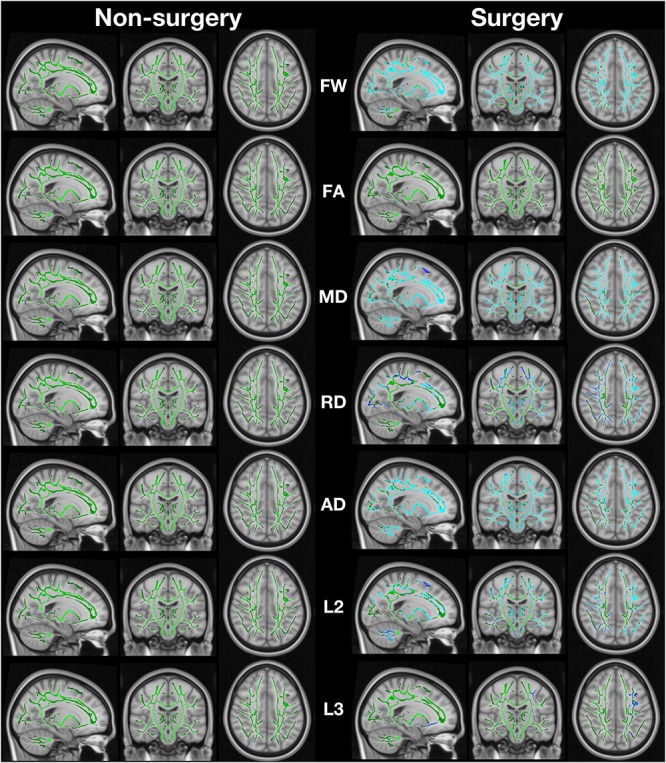
Image shows regions of change in fractional anisotropy (FA), mean diffusivity (MD), radial diffusivity (RD; calculated as the mean of λ_2_ and λ_3_), axial diffusivity (AD; calculated as λ_1_), λ_2_ (L2), and λ_3_ (L3). The green regions are the white matter skeleton created by TBSS. Blue indicates regions where post > pre. Non-surgery participants had no areas of significant change while surgery participants had extensive change (FSL *randomise* paired *t*-test with 5000 permutations).

When comparing post-surgery to pre-surgery, the TKA group had significant widespread increase in FW, MD, AD (λ_1_), RD, λ_2_, and λ_3_ (family-wise error corrected *p* < 0.05; Figure 1). There were no significant differences in FA in the TKA group. Non-TKA peers demonstrate no pre-post differences in any metric. As a *post hoc* analysis, we performed between-group TBSS analyses at each timepoint. There were no pre-surgery baseline differences in FW or any DTI metric. At the post-surgery timepoint, the TKA group had higher FW than the non-TKA group with the widespread pattern similar to the within-TKA group analyses. The primary metric of interest for individual-level analyses is FW but MD, AD, and RD were also assessed to investigate relationship between baseline participant characteristics and perioperative white matter structural changes. The individual with the acute stroke was not an outlier on any of the metrics (all values were within the interquartile range of the post-surgery group).

### Cognition and Brain Predictors of Free-Water Change

Age had a positive association with pre-surgical FW (*p* = 0.034) but no significant relationships with race or any other free-water or DTI metrics (Table 2). Race did not associate with any independent predictors. Other relationships between pre-surgical free-water and associated metrics and metric changes are presented in Table 2.

**TABLE 2:** Correlations among and descriptive statistics for demographic and dependent variables.

	Age	Race	PreFW	PreMD	PreAD	PreRD	Post-Pre	Post-Pre	Post-Pre	Post-Pre
							FW	MD	AD	RD
Age	1	0.03	$0.27^{*}$	0.16	0.22	0.04	0.02	-0.12	-0.08	-0.09
Race		1	-0.13	-0.11	-0.13	-0.06	0.11	0.09	-0.04	0.09
PreFW			1	$0.79^{**}$	$0.33^{**}$	$0.76^{**}$	-0.23	$-0.48^{**}$	$-0.34^{**}$	$-0.44^{**}$
PreMD				1	$0.58^{**}$	$0.92^{**}$	-0.19	$-0.68^{**}$	$-0.43^{**}$	$-0.63^{**}$
PreAD					1	0.24	0.06	$-0.33^{**}$	$-0.60^{**}$	-0.13
PreRD						1	-0.24	$-0.65^{**}$	-0.24	$-0.70^{**}$
Post-Pre FW							1	$0.65^{**}$	0.22	0.63
Post-Pre MD								1	$0.57^{**}$	$0.90^{**}$
Post-Pre AD									1	0.19
Post-Pre RD										1

*N* = 61. PreFW, Pre-surgical free-water; PreMD, Pre-surgical mean diffusivity corrected for free-water; PreAD, Pre-surgical axial diffusivity corrected for free-water; PreRD, Pre-surgical radial diffusivity corrected for free-water; Post-Pre FW, Post-surgical free-water – pre-surgical free-water; Post-Pre MD, Post-surgical mean diffusivity corrected for free-water – pre-surgical mean diffusivity corrected for free-water; Post-Pre AD, Post-surgical axial diffusivity corrected for free-water – pre-surgical axial diffusivity corrected for free-water; Post-Pre RD, Post-surgical radial diffusivity corrected for free-water – pre-surgical radial diffusivity corrected for free-water. *p < 0.05. **p < 0.01.

The results of the hierarchical regression predicting free-water change from cognitive and brain variables are presented in Table 3. Frontal-subcortical function was the only significant predictor (*p* = 0.01; medium effect size) of free-water change with current brain integrity (BI) suggestive at a trend level (*p* = 0.07; small effect size); higher scores resulted in greater free-water increase after surgery. Age, race, cognitive reserve (CR), and delay memory (DM) were not significant predictors of free-water change (*p*-values > 0.179). These regression models did not significantly predict MD change (*F* = 1.32, *p* = 0.266), AD change (*F* = 0.57, *p* = 0.750), or RD change (*F* = 1.16, *p* = 0.341).

**TABLE 3:** Summary of hierarchical regression analysis for variables predicting free-water change (*N* = 61).

	Model 1			Model 2			Model 3			Model 4			Model 5			Model 6		
Variable	*B*	*SE(B)*	*β*	*B*	*SE(B)*	*β*	*B*	*SE(B)*	*β*	*B*	*SE(B)*	*β*	*B*	*SE(B)*	*β*	*B*	*SE(B)*	*β*
Age	4.77⁢e-5	0.000	0.020	3.91⁢e-5	0.000	0.016	0.001	0.000	0.226	0.000	0.000	0.192	0.000	0.000	0.169	0.000	0.000	0.173
Race	0.005	0.006	0.106	0.005	0.006	0.111	0.009	0.006	0.181	0.013	0.007	$0.256^{\wedge }$	0.013	0.006	$0.256^{\wedge }$	0.012	0.006	$0.242^{\wedge }$
CR				0.002	0.003	0.076	-0.001	0.004	-0.035	-0.003	0.004	-0.117	-0.007	0.004	$-0.252^{\wedge }$	-0.007	0.004	$-0.247^{\wedge }$
BI							0.008	0.004	$0.371^{*}$	0.008	0.004	$0.345^{*}$	0.007	0.004	$0.306^{\wedge }$	0.008	0.004	$0.331^{*}$
PS										0.008	0.004	$0.271^{\wedge }$	0.006	0.004	0.205	0.007	0.004	0.223
IM													0.008	0.003	$0.355^{*}$	0.010	0.004	$0.444^{**}$
DM		0.012			0.018			0.095								-0.004	0.003	-0.169

*R^2^*		0.347			0.332			$4.760^{*}$			0.152			0.244			0.260	
*F* for change in *R^2^*											$3.711^{\wedge }$			$6.553^{*}$			1.148	

CR, cognitive reserve; BI, brain integrity; PS, processing speed; IM, immediate memory; DM, delay memory. *p < 0.05. **p < 0.01. ${}^{\wedge }$0.05 < *p* < 0.10.

## Discussion

Free-water imaging, a type of DWI analysis, can provide information regarding water diffusivity in tissue and extracellular components. It is proposed extracellular free-water volume increases with neuroinflammation (Pasternak et al., 2009, 2012, 2015), although it could increase due to other factors such as neurodegeneration (Ofori et al., 2015). Within 48 h of surgery and anesthesia, TKA participants’ white matter showed increases in free-water, mean diffusivity, axial diffusivity, and radial diffusivity. The free-water findings indicate increased white matter extracellular volume. We speculate this finding represents widespread neuroinflammation. Axonal compression and decreased axonal diameters driven by increased extracellular volume could explain the MD and AD findings. It is possible, however, that MD, AD, and RD changes were secondary effects or artifacts of free-water modeling algorithms. Even though we demonstrated MD, AD, and RD changes, the absence of FA changes suggests little or no acute neuronal damage.

Importantly, we show individuals with higher frontal-subcortical functions and brain integrity *prior* to TKA with general anesthesia had greater increases in free-water throughout the core brain white matter. Taken together, while the specific etiology of free-water increases is unknown, a possible explanation for the results is an active cerebral inflammatory response. If the free-water increase represents an active cerebral inflammatory response, individuals with higher preoperative cognition and greater brain integrity appear to have more resilience to cope with acute cerebral insult via immunological mechanisms. If there is a dominant insulting agent remains an unknown, but we hypothesize the combined effects of anesthetic agents, altered cerebral perfusion, acute pain, surgical stress, and other perioperative and postoperative factors all contribute to extracellular white matter changes.

### Chronic Versus Reactive Inflammation?

One possible interpretation of these results is a responsive glial activation, an acute inflammatory state distinct from - if not completely independent from – chronic neuroinflammation or neurodegeneration. Chronic neuroinflammation is linked with Alzheimer’s disease (AD), Parkinson’s disease, and multiple sclerosis (MS) (see for review Heneka et al., 2015; Ransohoff, 2016). In AD, for example, astrocytes and microglia appear to interact in a complex way with neuropathology, blood-brain barrier disruption, or other systemic changes in the parenchymal environment (Heneka et al., 2015). While the deleterious effects of chronic inflammation in the brain are becoming clearer, in contrast, less is known about active (reactive) inflammatory states in the human brain, particularly in the context of major surgery.

Notably, non-demented older adults with mild current cognitive inefficiencies and reduced brain integrity have restricted neuroimaging free-water change (Reas et al., 2018). This research suggests cognitive and brain integrity may predict glial response. If free-water increase is a marker for reactive inflammation (glial response), then individuals with *reduced* preoperative cognitive and brain integrity electing surgery may have a muted response and possibly reduced cerebral “wound” care.

Previous research indicates reactive glial states might be harmful, neutral, or beneficial depending on location, severity, and chronicity (Ransohoff, 2016). There is *in vitro* evidence pentobarbital results in swollen, “active” microglia (Kannan et al., 2017). It is possible other anesthetics similarly affect glial cells, creating or adding to a reactive neural inflammatory state. However, in TBI, propofol appears to limit microglial activation, which might serve a neuroprotective function for cognition (Luo et al., 2013). Similarly, in animal models of surgery, elevated interleukin-1β (IL-1β) is associated with memory dysfunction; pretreatment with an IL-1 receptor antagonist reduced neuroinflammatory effects and memory dysfunction (Cibelli et al., 2010). In contrast, however, it was shown in AD that greater baseline (acute/reactive) microglial activation as measured through ^18^F-DPA-714 binding was associated with better clinical prognosis 2 years later. Individuals with lower baseline microglial activation subsequently had higher (chronic) microglial activation and worse clinical outcome (Hamelin et al., 2018). While we do not know if the free-water changes in the context of major surgery indicate beneficial neuroinflammation, or are specific to inflammation, it is suggestive that those with worse cognition and lower brain integrity have a muted perioperative response. We speculate those who have muted responses are less able to return to free-water baseline and the acute inflammation increases chronic inflammation. Additionally, those who have worse baseline cognition and brain integrity are at increased risk of adverse outcomes after major surgery (Chen et al., 2014; Price et al., 2014; Culley et al., 2017) so subdued acute postoperative neuroinflammation might be a mechanism for postoperative cognitive changes. This is an area for future investigation.

Patients with mild cognitive impairment (MCI) who are electing surgery might have chronic inflammation and altered (senescent) microglia, thereby resulting in a restricted ability to respond to neural insults associated with surgery and general anesthesia. While we did not analyze MCI, it is possible MCI could partially explain the lower levels of free-water increase after surgery. Importantly, our results also suggest a potential mechanism for neurodegeneration in the future. If major surgery under general anesthesia acts as a mild but widespread brain trauma with associated innate immune response, it is possible the initial healing response will interact with existing neuropathology, weakened blood-brain barriers, and chronic inflammation to result in future additional pathological burden and neurodegeneration. Research suggests undergoing anesthesia and surgery result in increased risk of dementia and reduced time to dementia diagnosis (Chen et al., 2014). However, a study with 22 female patients using cerebrospinal fluid markers of inflammation indicated that while a neuroinflammatory response occurs there were not significant beta-amyloid or tau biomarker changes (Pikwer et al., 2017). Those results were preliminary and do not rule out a subtle longitudinal cascading effect on neuropathology. They do, however, support an interpretation of our results as indicating a reactive glial state following surgery.

Hirsch et al. (2016) assessed cerebrospinal fluid (CSF) and plasma inflammatory markers after orthopedic surgery in a pilot group of 11 older patients. They found increased pro- and anti-inflammatory activity in the central nervous system after surgery. Intriguingly, Hirsch et al. (2016) had preliminary data suggesting patients with acute postoperative cognitive decline had lower preoperative levels of some pro and anti-inflammatory plasma cytokines with some cytokines continuing to remain lower after surgery. In contrast, some CSF cytokines were higher preoperatively and remained higher postoperatively in patients who developed cognitive difficulties acutely after surgery. The mixed results are likely cytokine-specific and could relate to timing of measurements. In other words, reactive glial states might be harmful, neutral, or beneficial depending on a number of factors including how, what, and when measurements are taken (Ransohoff, 2016).

### Glymphatic System

An alternative but not necessarily competing explanation for free-water increases relates to the glymphatic system – a system believed to play a role in the clearance of cerebral waste (Iliff and Nedergaard, 2013). Neural injury results in increased generation of metabolic byproducts. Major surgery under general anesthesia might result in transient neural injury, at least for older adults. There would then be a need for the glymphatic system to activate and clear excessive metabolic byproducts. Evidence suggests the glymphatic system is more active during sleep and under general anesthesia (Xie et al., 2013). During sleep and under anesthesia there is increased interstitial fluid volume associated with activation of the glymphatic system. It is believed sleep might thus serve in part as a restorative function to remove accumulation of potentially neurotoxic waste products (Xie et al., 2013).

Apart from sleep, if general anesthesia also results in increased interstitial fluid volume (Xie et al., 2013), increased free-water in our participants could be caused by increased interstitial fluid volume secondary to general anesthesia. The relationship between interstitial fluid volume and anesthesia is not clear, however, in light of contradictory findings by Gakuba et al. (2018) who did not find increases in extracellular water volume in anesthetized mice.

There is indirect evidence in favor of a glymphatic hypothesis for why patients with reduced cognitive function may have less pre-post microstructural free-water change. Tarasoff-Conway et al. (2015) provide a review of research implications of brain clearance systems for Alzheimer’s disease. *Aqp4* knockout mouse models demonstrate reduced interstitial and Aβ clearance. Additionally, mouse models of traumatic brain injury suggest impaired glymphatic function for at least one-month post-injury (Iliff et al., 2014). Individuals with accumulating Aβ and disrupted interstitial clearance, which might be the case in individuals who have prodromal Alzheimer’s disease, thus might be less likely to have a responsive glymphatic system and less extracellular volume increase (Kress et al., 2014).

While the etiology of the free-water increases is unknown, we observed extensive acute white matter free-water changes after major orthopedic surgery. Of the two explanations explored, both suggest glial involvement. There are other possibilities, but there appears to be a widespread glial activation after major TKA with general anesthesia. While most participants experienced free-water increase, those who had greater immediate memory and brain integrity had relatively greater acute perioperative increases. Therefore, if the free-water change represents glial activation, our results suggest the reactive glial response is greater in individuals with higher scores on preoperative measures of immediate memory and brain integrity. Whether or not free-water change has longitudinal benefits for cognition and recovery is under investigation. Remaining unknowns also include (1) the specific etiology of changes, (2) if diffusion changes persist after the acute postoperative stage, (3) if acute white matter changes are predictive of other later adverse events, and (4) if mild cognitive impairment as a classification is predictive of restricted free-water changes.

### Other Considerations

There are other considerations for etiology behind extracellular free-water changes. Widespread edema secondary to blood brain barrier disruption is a possibility; cerebral edema has been shown acutely after major cardiac surgery (Harris et al., 1993; Anderson et al., 1999). This could be an area of focus in future analyses of the etiology of extracellular free-water increases after major surgery.

One of the components of our brain integrity composite was leukoaraiosis, which is associated with cerebrovascular and cardiovascular disease. Cardiovascular disease might play a mechanistic role in free-water change after surgery. However, in a *post hoc* analysis found no significant correlations between FW change and other cardiovascular study variables (i.e., systolic and diastolic blood pressure, pulse pressure, cholesterol, triglycerides, HDL, LDL, self-reported cardiovascular risk factors, *p*-values > 0.233).

### Limitations

We did not acquire postoperative IL-6, TNF-alpha, CRP or other plasma or CSF markers of inflammation. Blood markers, however, are non-specific to neuroinflammation so, while they might provide evidence of inflammation, without corroborating markers of neuroinflammation (e.g., free-water increase) they are not sufficient markers of neuroinflammation. While we are not certain the changes in free-water result from neuroinflammation, free-water change is localized within the brain and are a mechanism and method worth continued investigation. Another limitation is we did not acquire a repeated FLAIR scan to quantify LA changes, which might have yielded additional information about acute white matter changes. We also did not assess free-water changes within gray matter regions. Gray matter free-water change analyses are encouraged in future research.

## Conclusion

We demonstrated older adults undergoing TKA under general anesthesia have extensive increases in white matter free-water, suggestive of extracellular volume increases. Importantly, older adults with better frontal-subcortical cognitive functions (e.g., immediate memory) and better brain integrity had relatively larger increases in free-water. Our results suggest an active white matter glial response (e.g., neuroinflammation and/or glymphatic changes) to surgery under general anesthesia.

## Ethics Statement

This study was carried out in accordance with the recommendations of the University of Florida Institutional Review Board in Gainesville, Florida with written informed consent from all subjects. All subjects gave written informed consent in accordance with the Declaration of Helsinki. The protocol was approved by the University of Florida Institutional Review Board.

## Author Contributions

CCP, HP, and TM contributed to the design of the research. JT, HP, and CCP contributed to the implementation of the research. JT, MA, CH, DV, TM, and CCP contributed to the analysis or interpretation of the results. JT, MA, HP, DV, and CCP contributed to the writing of the manuscript.

## Conflict of Interest Statement

The authors declare that the research was conducted in the absence of any commercial or financial relationships that could be construed as a potential conflict of interest.

## Footnotes

1 http://surfer.nmr.mgh.harvard.edu/

2 https://imagej.nih.gov
